# Effects of Defocus Distance and Weld Spacing on Microstructure and Properties of Femtosecond Laser Welded Quartz Glass-TC4 Alloy Joints with Residual Stress Analysis

**DOI:** 10.3390/ma18143390

**Published:** 2025-07-19

**Authors:** Gang Wang, Runbo Zhang, Xiangyu Xu, Ren Yuan, Xuteng Lv, Chenglei Fan

**Affiliations:** State Key Laboratory of Precision Welding & Joining of Materials and Structures, Harbin Institute of Technology, Harbin 150001, China

**Keywords:** femtosecond laser, quartz glass, TC4 alloy, residual stress

## Abstract

This study develops an optimized femtosecond laser welding process for joining quartz glass and TC4 titanium alloy (Ti-6Al-4V) under non-optical contact conditions, specifically addressing the manufacturing needs of specialized photoelectric effect research containers. The joint primarily consists of parallel laser-welded zones (WZ) interspersed with base material. The defocus distance of the femtosecond laser predominantly influences the depth and phase composition of the WZ, while the weld spacing influences the crack distribution in the joint region. The maximum shear strength of 14.4 MPa was achieved at a defocusing distance of +0.1 mm (below the interface) and a weld spacing of 40 μm. The XRD stress measurements indicate that the defocusing distance mainly affects the stress along the direction of laser impact (DLI), whereas the weld spacing primarily influences the stress along the direction of spacing (DS). GPA results demonstrate that when the spacing is less than 30 μm, the non-uniform shrinkage inside the WZ induces tensile stress in the joint, leading to significant fluctuations in DS residual stress and consequently affecting the joint’s shear strength. This study investigates the effects of process parameters on the mechanical properties of dissimilar joints and, for the first time, analyzes the relationship between joint residual strain and femtosecond laser weld spacing, providing valuable insights for optimizing femtosecond laser welding processes.

## 1. Introduction

The joining of dissimilar materials is a key focus and challenge in materials science and engineering. By combining materials with different surface states, strengths, and physical/chemical properties, their performance advantages can be fully utilized while reducing costs, achieving a synergistic “1 + 1 > 2” effect [1,2]. Specifically, transparent ceramics generally exhibit excellent optical transparency and wear resistance. If they can be successfully joined with metals possessing high strength and toughness, it would enable the fabrication of devices that integrate both optical and mechanical properties [3,4]. Currently, a specialized container for studying the photoelectric effect is under development, using quartz glass and TC4 titanium alloy (Ti-6Al-4V) as the selected materials.

However, joining transparent ceramics and metals presents significant challenges. From a structural perspective, ceramics are primarily composed of covalent/ionic bonds, whereas metals consist of metallic bonds and free electrons. This difference in bonding mechanisms makes it difficult for their atoms to diffuse into each other’s lattice structures to form a metallurgical bond [5]. From a physical property standpoint, the thermal expansion coefficient (CTE) of metals is often ten times higher than that of ceramics. If the welding temperature is too high or the heat input is uneven, thermal mismatch-induced cracking will occur at the joint interface [6]. To overcome this difficulty, non-fusion bonding methods such as vacuum brazing and ultrasonic brazing are often employed, yet they still suffer from issues like low processing efficiency and poor joint performance. 

Femtosecond laser welding is an emerging specialized welding technology that first appeared in 2005 [7] and has gradually become commercialized in recent years [8,9,10]. Due to the nonlinear absorption effect of femtosecond laser in transparent materials, multiphoton ionization occurs [11,12,13] at the glass-metal interface, generating plasma and forming a molten pool of sufficient volume [14,15,16], thereby achieving metallurgical bonding between the two materials. The efficiency of the nonlinear absorption effect is highly dependent on the laser energy density [17,18]. According to the literature, the energy density at the femtosecond laser focal point exceeds that of picosecond lasers by more than fivefold, and the advantage becomes even more pronounced when compared to nanosecond lasers [19]. As a result, femtosecond lasers enable high-strength bonding between quartz glass and TC4 titanium alloy at relatively low power levels. [20,21]. Leveraging these characteristics, femtosecond laser welding enables high-efficiency and the low-damage bonding of glass and metal without requiring an intermediate layer, facilitating lightweight device production [22,23].

The joining of quartz glass and TC4 titanium alloy constitutes a bonding between amorphous and crystalline materials. Through precise control of process parameters to modulate laser heat input, interfacial fusion can be achieved, thereby overcoming the inherent structural incompatibility between these dissimilar materials. However, the significant toughness disparity (quartz glass being brittle versus ductile TC4 alloy) necessitates careful heat input management, as excessive thermal energy may induce cracking in the glass due to thermal shock. Consequently, meticulous optimization of femtosecond laser parameters becomes imperative for obtaining high-performance joints.

Femtosecond laser dissimilar welding has been successfully applied to various types of glass and metals. Ozeki et al. [24] achieved the first direct glass-to-metal welding using a femtosecond laser in 2008. The bonding of metal and glass materials results from the combined effects of linear and nonlinear laser absorption. Fujiwara et al. [25] also successfully sealed a glass–metal-structure device using a 250 fs pulse laser. Zhan et al. [26] successfully performed femtosecond laser welding of quartz glass and pure aluminum, utilizing high-speed photography to observe the welding process. By comparing plasma morphologies under different process parameters, the research team optimized the welding process and found that the joint exhibited the best mechanical properties when the defocus amount was **+** 0.08 mm. These studies demonstrate that high pulse energy welding enhances joint strength and expands the element mixing regions. Our previous work [27] elucidated the influence of femtosecond laser pulse energy on the microstructure and mechanical properties of quartz glass-TC4 welded joints, and through process parameter optimization, achieved a joint with a shear strength of 10.4 MPa under non-optical contact conditions.

Given the small molten pool volume in femtosecond laser welding, welding paths are typically used to fill the bonding area to ensure sufficient effective welding area and joint strength. In addition to parameters such as laser power and defocus amount, the spacing between welding paths is also a critical factor. Since glass generally exhibits lower ductility and thermal shock resistance than metals, inappropriate defocus amounts and welding spacings may induce stress concentration at the joint, reducing shear strength and device performance. For stress analysis in welded joints, the most common method is the deep-hole drill (DHD) technique, a destructive testing approach that requires drilling small holes in the base material, which may damage quartz glass and lead to joint failure. X-ray diffractometer (XRD) stress measurement, on the other hand, is a non-destructive testing method that utilizes Bragg diffraction between X-rays and crystal lattices to determine stress magnitude and direction in joints [28].

This paper investigates the effects of defocus amount and welding spacing on joint microstructure and performance, further optimizing the shear strength of quartz glass-TC4 welded joints based on preliminary work. Additionally, XRD and geometric phase analysis (GPA) are employed to analyze residual stress distribution in the joints, providing a theoretical foundation for process optimization.

## 2. Materials and Methods

### 2.1. Base Materials and Welding Method

**Figure 1:**
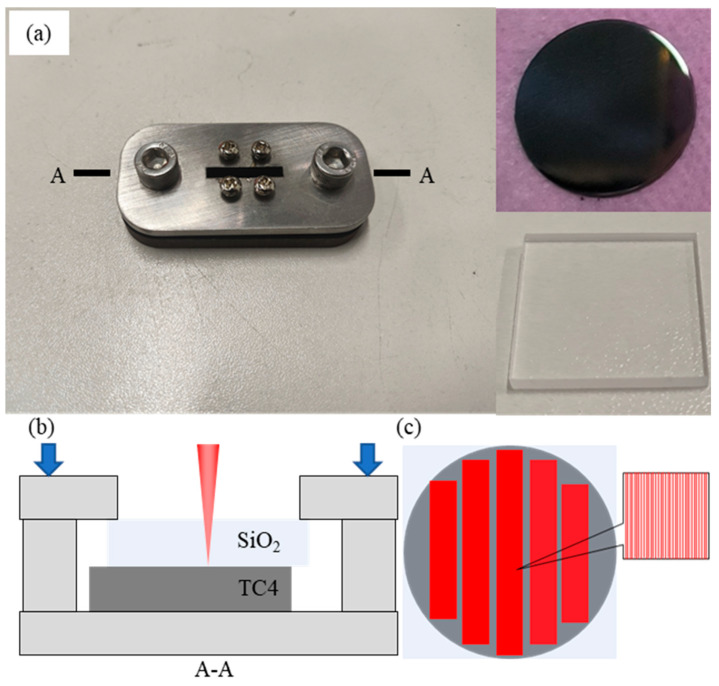
(**a**) The weld clamp and BMs, (**b**) schematic diagram of the welding process, (**c**) femtosecond laser weld paths. The section plane A-A in (**a**) is displayed in (b).

A quartz glass piece (20 × 20 × 1 mm^3^) was placed on a TC4 disc (20 mm diameter, 1 mm thickness) with a surface roughness of about Ra 1 μm as base materials (BMs). The chemical composition of the BMs is listed in Table 1. Both are placed on a clamp and stacked with a pressure of 85~95 kPa, as shown in Figure 1a,b. The welding path is illustrated in Figure 1c, where parallel linear weld paths were employed for transmission welding. In previous studies [27], perpendicular linear weld paths were utilized to enhance joint bonding strength. To prevent complex stress distribution arising from multi-directional welding patterns, a unidirectional parallel welding strategy was adopted in this study.

The welding process was carried out using a femtosecond laser device from Nanjing Keyun Laser Co., Ltd. (Nanjing, China). This device has a rated power of 20 W, with a minimum pulse width of 300 fs and a focal diameter of 26 μm. Based on previous optimized parameters (laser power of 3 W, pulse frequency of 10 kHz, and welding speed of 50 mm/s) that considered material absorptivity and physical properties [27] to produce robust joints, this work intends to examine how defocusing distance and weld spacing influence joint microstructure and performance.

**Figure 2:**
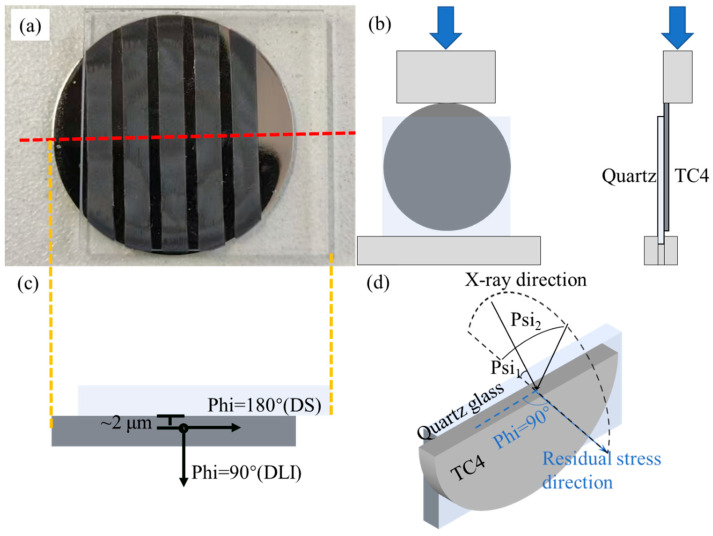
(**a**) Joint cross-section sampling location (red dashed line), (**b**) shear testing methodology, (**c**) XRD-RSM measurement positions, (**d**) a 3D schematic diagram of XRD-RSM process. The yellow dashed line denotes longitudinal alignment between (a) [top view] and (c) [front view].

The experimental parameters used for welding are listed in Table 2. Note that a negative/positive defocus distance indicates a laser focal plane is positioned above/below the material interface. Each group of parameters was tested four times to ensure the accuracy and repeatability of the test results. After the welding experiments, welded area and weld formation of the first specimen corresponding to each group was observed using a KEYENCE VHX 6000 extended depth-of-field optical microscope (Osaka, Japan), and the average shear strength of another three specimens was taken as the mechanical test result. The first specimen was then cut perpendicular to the weld paths, as shown in Figure 2a, and the method of shear strength test was shown in Figure 2b. The cross-sectional specimens were then used for scanning electron microscope (SEM) observation, XRD residual stress measurement (XRD-RSM), high-resolution transmission microscope (HRTEM) observation, and geometrical phase analysis (GPA). The SEM observation was completed using a TESCON SOLARIS 2 SEM (Brno, Czech Republic) device, with an operating voltage of 20 kV. All SEM specimens were ground with metallographic sandpaper and polished with diamond abrasive prior to observation. The shear strength of the joints was tested using an Instron 5988 electronic universal testing machine (Marshall, MI, USA), with the testing procedure shown in Figure 2. The loading speed used during the test was 0.5 mm/min.

### 2.2. XRD-RSM

XRD-RSM determines the internal stress in materials by measuring variations in interplanar spacing based on Bragg’s law and elastic mechanics theory. The underlying principle can be explained by Equation (1) [28]:

*d*_(*Psi*)_ = *d*_0_[1 + (1 + *ν*)*σ*_(*Phi*)_sin^2^*(Psi)*/E] 1 (1)

where *σ*_(*Phi*)_ represents the residual stress along the Phi-direction; *d*_(*Psi*)_ denotes the interplanar spacing along the Psi-direction, measured by X-ray diffraction with incident beams parallel to this direction; *d*_0_ refers to the reference interplanar spacing in a stress-free state; while *ν* and E represent the Poisson’s ratio and Young’s modulus of the base material, respectively.

The measurement locations and stress directions for XRD-RSM analysis are illustrated in Figure 2c,d. Stress measurements were conducted at a depth of approximately 2 μm in the TC4 BM using an X’Pert Pro X-ray diffractometer (Malvern Panalytical) (Amsterdam, Netherlands). The interplanar spacing *d*_(*Psi*)_ was measured at varying *Psi* angles, and the corresponding *d*_(*Psi*)_ vs. sin^2^*(Psi)* curves were plotted. The residual stress *σ*_(*Phi*)_ along specific directions was then calculated by determining the slope of these curves and substituting it into Equation 1.

The XRD measurements were performed using a Cu Kα radiation source (λ = 1.5406 Å) with the following acquisition parameters: a narrow 2θ range of 130–134° was carefully selected to target specific diffraction peaks, while employing a fine step size of 0.03° to ensure high angular resolution. Two principal stress directions were investigated:

1. The direction of laser impulse (DLI, Phi = 90°),
2. The direction of spacing (DS, Phi = 180°).

### 2.3. HRTEM Observasion and GPA

To investigate the micro-scale strain distribution in the welded region, TEM was employed for imaging, followed by GPA using the Digital Micrograph software suite (v. 3.53.4137.0). Site-specific milled foils for TEM observation were prepared from the weld zone (WZ) using a focused ion beam (FIB) system integrated with the SEM setup. A region located 2 μm from the interface was selected for FIB milling, and the thickness of milled sample was 60–80 nm. TEM characterization was performed using a Tecnai G2 F30 microscope (FEI Company) (Hillsborough, OR, USA) with a beam voltage of 300 kV. The GPA plugin enabled quantitative computation of strain fields from acquired high-resolution TEM (HRTEM) images and their corresponding fast Fourier transform (FFT) patterns. Then two-dimensional strain distribution maps along specified crystallographic directions will be generated. For the present study, the strain components were defined as follows:

*Exx*: Strain along DLI,

*Eyy*: Strain along DS.

## 3. Results and Discussion

### 3.1. Microstructure

**Figure 3:**
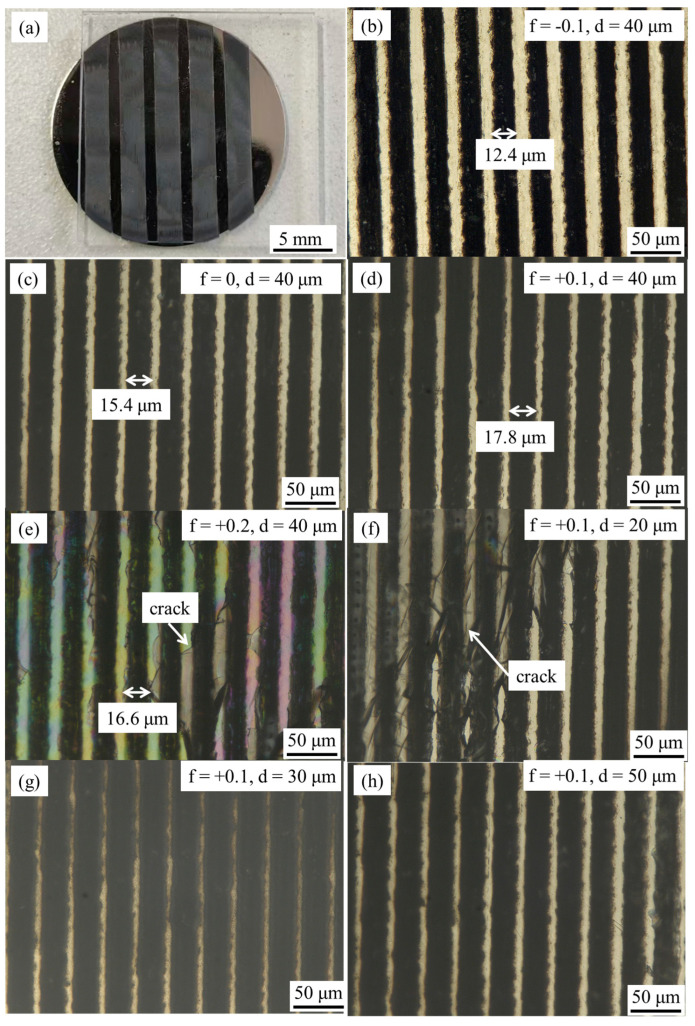
(**a**) Top view of femtosecond-welded SiO_2_-TC4 joint, and magnified image of the weld area with parameters of (**b**) f= − 0.1 mm, d = 40 μm, (**c**) f = 0 mm, d = 40 μm, (**d**) f = + 0.1 mm, d = 40 μm, (**e**) f = + 0.2 mm, d = 40 μm, (**f**) f = + 0.1 mm, d = 20 μm, (**g**) f = + 0.1 mm, d = 30 μm, (**h**) f = + 0.1 mm, d = 50 μm.

Figure 3a shows a femtosecond laser welding specimen of quartz glass-TC4, with + 0.1 mm defocus distance and 40 µm weld spacing, where the quartz glass retained good optical transparency after welding. The WZ exhibits uniform filling by weld lines without visible cracks or welding defects. Due to the extremely small spot diameter of the femtosecond laser, optical microscopy was employed to characterize the top-view of the welds, as shown in Figure 3b–h, corresponding to the joints of group 1 to group 7, respectively. The joint primarily consists of parallel laser-welded zones (dark region) interspersed with BMs (light region). At negative defocus distances (Figure 3b), the limited energy deposition in TC4 base metal (BM) resulted in narrow weld lines (~12.4 μm width). With increasing defocus distance (focal plane moving downward), enhanced energy absorption by TC4 BM progressively widened the weld lines. Notably, cracks occurred at + 0.2 mm defocus distance (Figure 3e), attributed to thermal mismatch (16× difference in thermal expansion coefficients between TC4 and quartz glass) induced by the differential laser energy absorption of two BMs. Similar cracks appeared in specimens with 20 μm weld spacing, as shown in Figure 3h. This phenomenon arises from micro-deformations generated during the melting and solidification of base metals. When the weld line spacing is too small (e.g., 20 μm), the strain fields of adjacent weld lines interact synergistically, leading to localized stress concentration. The resulting stress accumulation exceeds the fracture toughness of the interfacial region, thereby initiating cracks.

**Figure 4:**
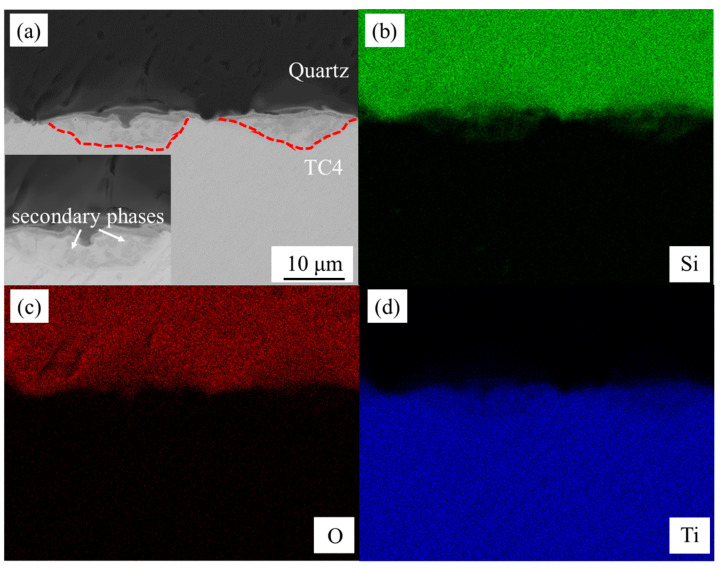
(**a**) A BSE-SEM image of the weld cross-section with +0.1 mm defocus distance and 40 µm weld spacing, along with the microstructure of a WZ, (**b**) element distribution map of Si, (**c**) element distribution map of O, (**d**) element distribution map of Ti.

The joint interface produced at + 0.1 mm defocus distance was characterized using SEM in backscattered electron (BSE) mode as shown in Figure 4a, revealing discontinuous spindle-shaped WZs between quartz glass (upper BM) and TC4 titanium alloy (lower BM), as marked with red dashed lines. Secondary phases were observed near the TC4 interface, indicating interfacial reactions during welding. According to the literature [29], such glass–metal femtosecond laser joints exhibit hybrid mechanical–chemical bonding, featuring metallurgical bonding within WZs and van der Waals forces at WZ/BM interfaces. EDS elemental mapping (Figure 4b–d) confirmed interdiffusion of Si, O (from glass), and Ti (from TC4) throughout the joint, with Si distribution precisely matching the WZ boundary (red dashed line in Figure 4a), demonstrating complete elemental mixing during the welding process.

**Figure 5:**
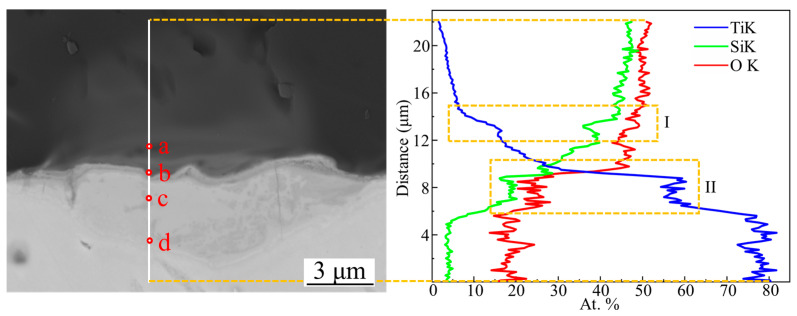
EDS linear scanning result of the white line, and four spots of EDS point analysis.

The EDS line scanning results across the WZ are presented in Figure 5, with the white line indicating the scanning path. A notable sharp increase in Ti content is observed in two distinct regions: Zone I located within the quartz base material (BM), suggesting the formation of Ti-containing compounds; and Zone II at the interface between the base materials, which exhibits a significant compositional gradient extending approximately 2 μm, indicating the presence of a well-defined interfacial reaction layer.

Point analysis conducted at specific locations along the scanning path, as shown in Table 3, reveals that point a in Zone I consists primarily of SiO_2_ and TiSi_2_, while point b at the interface contains a mixture of SiO_2_, TiSi_2_, and elemental Ti. Point c contains higher Si content compared with point d, suggesting a greater concentration of TiSi_2_. The line scanning profiles further demonstrate that Si exhibits weaker diffusion compared to O [30], as evidenced by its significantly lower content in the TC4 base material and non-uniform distribution within the melt pool. This inhomogeneous Si distribution, along with the formation of dark secondary phases, reflects localized segregation of Si and its subsequent reaction with Ti to form TiSi_2_.

**Figure 6:**
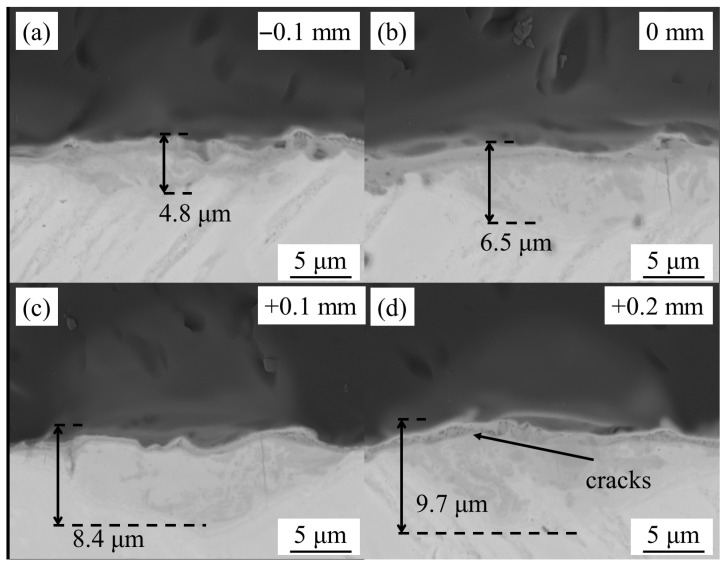
Microstructure of WZs with defocus distance of (**a**) −0.1 mm, (**b**) 0, (**c**) +0.1, (**d**) +0.2.

Figure 6 presents the microstructural evolution of WZs with different defocus distance, obtained by SEM in BSE mode. The primary effect of defocusing distance is manifested in the depth of the WZ. Under negative defocus conditions, minimal melting occurs in the TC4 BM, resulting in secondary phases densely concentrated at the interface. As the focal position moves downward, increased energy absorption by the TC4 BM leads to significant expansion in both width and depth of the WZ, corresponding to enhanced effective bonding area and improved mechanical properties.

**Figure 7:**
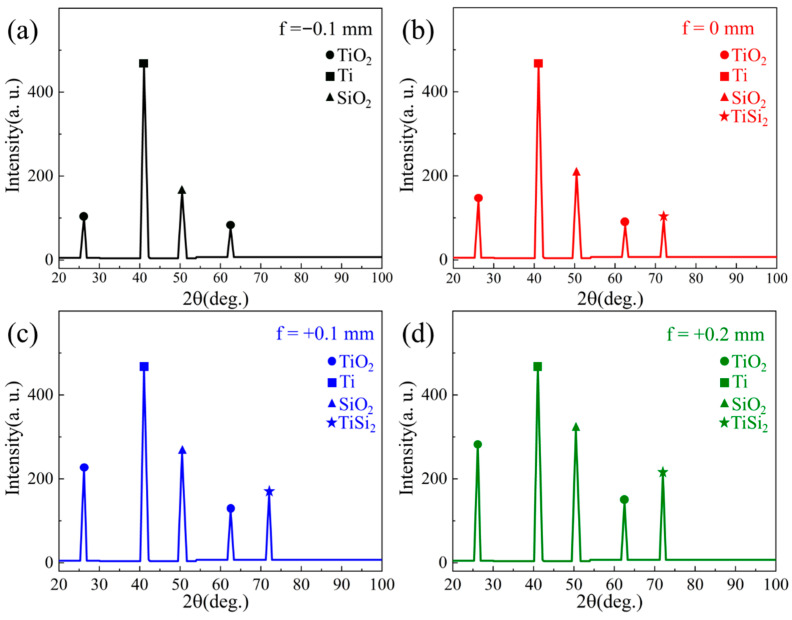
XRD phase composition analysis of joints with defocus distance of (**a**) −0.1 mm, (**b**) 0, (**c**) +0.1, (**d**) +0.2.

However, at a defocus distance of +0.2 mm, micro-cracks emerge at the interface due to thermal expansion of the TC4 BM exceeding the deformation tolerance of the quartz BM, ultimately degrading interfacial bonding strength. Phase composition analysis via XRD (Figure 7) was employed in micro-area scanning mode. The results reveal that joints produced with negative defocus contain trace amounts of TiO_2_. With a downward focal shift, TiSi_2_ becomes detectable alongside increased TiO_2_ content, demonstrating the progressive development of interfacial reactions with greater energy deposition in the TC4 BM.

### 3.2. Mechanical Properties

**Figure 8:**
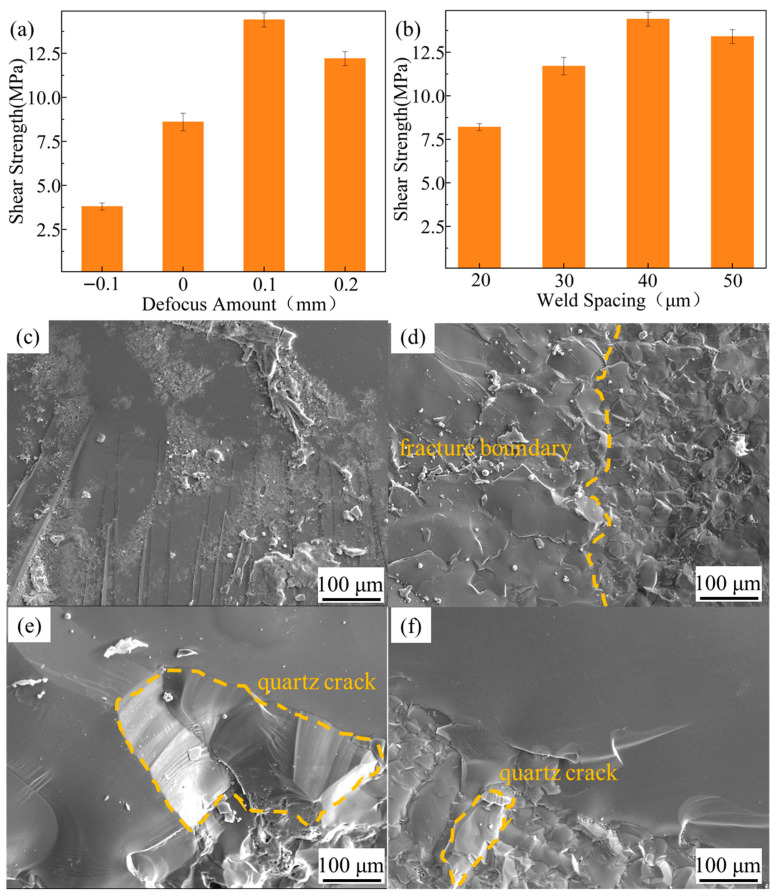
Joint shear strengths of (**a**) different defocus distances and (**b**) different weld spacing; (**c**–**f**) typical fracture surfaces of these joints.

Shear strength tests were conducted on welded joints fabricated with different process parameters. Figure 8a presents the shear strength of joints obtained at various defocusing distances. When the defocusing distance was negative, the shear strength was only 3.6 MPa. As the laser focal plane moved toward the TC4 base material (BM), the shear strength increased significantly. The maximum shear strength of 14.4 MPa was achieved at a defocusing distance of +0.1 mm. However, when the defocusing distance reached +0.2 mm, the shear strength decreased to 11.8 MPa. Figure 8b illustrates the shear strength of joints welded with different weld spacing. The specimen with weld spacing of 20 μm demonstrated significantly lower shear strength compared to the other three configurations, while the one with weld spacing of 40 μm exhibiting the highest strength of 14.4 MPa. Compared with the quartz glass/Al joints (8.94 MPa shear strength) reported by Zhang et al. [31], the present study achieved superior interfacial strength through optimized patterned welding that creates mechanical interlocking effects, combined with Ti’s higher reactivity and diffusivity in SiO_2_ than Al. The TC4 titanium alloy’s active Ti elements readily diffuse into quartz glass to form stronger Ti-Si-O metallurgical bonds, as confirmed by EDS and TEM analyses (Figure 5c and Figure 7d), demonstrating the advantages of both process innovation and material selection for high-performance glass–metal joining.

Typical fracture morphologies of these shear specimens were investigated subsequently. Figure 8c displays the fracture surface with a defocus distance of −0.1 mm, which appears smooth with minimal quartz adhesion on the TC4 surface, indicating insufficient fusion between the two BMs. This was attributed to the laser focal plane not reaching the TC4 surface and the blocking effect of quartz glass on the laser beam. According to the literature [32,33], the formation of transient plasma inside the glass by femtosecond laser irradiation resulted in low energy transmission and scattering at the interface. At this stage, the majority of thermal energy accumulates within the quartz base material (BM), while the heat in the TC4 BM primarily originates from thermal conduction through the molten quartz. Consequently, it becomes challenging to establish a WZ with optimal depth at the interface. Zhan et al. [26] quantitatively analyzed the relationship between femtosecond laser-induced plasma and energy utilization efficiency at the quartz glass/pure Al interface using high-speed photography. Their results revealed significant variations in plasma volume at the interface with changing defocus distances. When the defocus distance exceeded + 0.08 mm (consistent with the definition in this study), the laser energy could be sufficiently absorbed by the interfacial materials to form a stable molten pool, corresponding to optimal bonding strength at the interface. Similarly, fracture surfaces with defocus distances of 0, +0.1 mm, and +0.2 mm exhibited evident quartz glass adhesion and observable fracture boundaries between quartz BM and TC4 BM, as shown in Figure 8d. Notably, extensive cracking was observed in the quartz BM when defocus distance reached +0.2 mm or weld spacing was 20 μm, as shown in Figure 8e,f, suggesting fracture occurred within the quartz glass itself. Combined with the interfacial morphology shown in Figure 6d and shear test results, this phenomenon was attributed to thermal expansion of TC4 BM inducing interfacial cracks, and excessive stress concentration. According to Griffith Theory [34], such cracks increase the risk of brittle fracture even when external loads are below the fracture limit of quartz BM.

**Figure 9:**
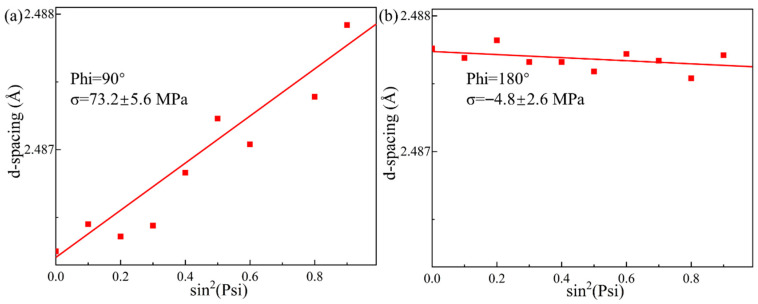
(**a**) DLI and (**b**) DS residual stress of the joint with defocus distance of +0.1 mm and weld spacing of 40 μm.

To further investigate residual stress distribution in femtosecond laser-welded joints, XRD-RSM tests were performed. The measurement point, located within the weld trajectory near the TC4 BM interface (Figure 2c), was analyzed. Figure 9a,b show the d(Psi) vs. sin^2^(Psi) curves obtained from XRD-RSM tests for the f = +0.1 mm, d = 40 μm specimen. According to Equation 1, DLI residual stress is 73.2 ± 5.6 MPa while DS residual stress is −4.8 ± 2.6 MPa. Subsequent measurements of other specimens (Table 4) revealed that defocusing distance primarily affected DLI residual stresses, attributable to the Gaussian heat source distribution model [35] where temperature peaks near the focal point in the DLI direction. Focal plane downward movement exacerbated thermal expansion of TC4 BM along the DLI direction, generating tensile stresses during non-uniform cooling. Weld spacing variations mainly influenced DS residual stresses due to uneven contraction between adjacent WZs. Notably, as the welding spacing decreased, the measurement error in residual stress significantly increased, indicating substantial fluctuations in microstrain within the WZ. This variability led to divergent results when X-ray scans were performed at different Psi angles.

**Figure 10:**
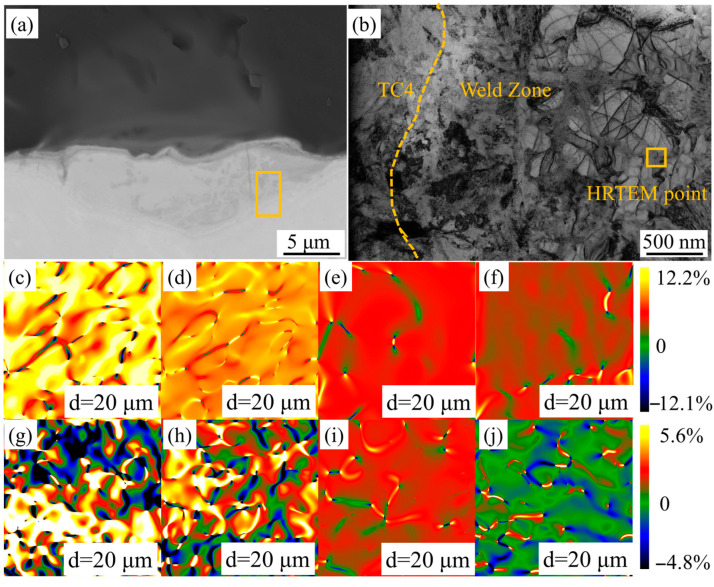
(**a**) FIB milling position, (**b**) TEM image of the FIB-processed sample and the HRTEM image of marked area, (**c**–**f**) GPA results in DLI, (**g**–**j**) GPA results in DS.

To investigate this strain fluctuation, FIB processing and TEM observation were conducted on the WZs of specimens with different welding spacings. A region located 2 μm from the interface was selected for FIB milling, as shown in Figure 10a, with the processed area measuring 5 μm × 3 μm. The post-processed specimen is shown in Figure 10b. HRTEM observation was performed on the WZ near the XRD-RSM measurement point, and images were processed using the GPA plugin, with the g-vector aligned parallel to the DLI direction, enabling the determination of microscopic strain distributions along both DLI and DS directions. The strain maps for specimens with different welding spacings are summarized in Figure 10c–j, where Figure 10c–f represents strain in the DLI direction and Figure 10g–j denotes strain in the perpendicular DS direction. The color-coded strain maps represent relative microstrain, with red/blue hues indicating tensile/compressive strains, respectively.

Analysis revealed that Exx exhibited predominantly tensile strain values, with magnitude increasing as welding spacing decreased. The Eyy strain behavior proved more complex: at d = 50 μm, overall compressive strain was observed, while at d = 40 μm, the strain became tensile. This phenomenon arises from the mutual influence of non-uniform contraction between adjacent WZs, making the resultant strain direction difficult to predict. For specimens with d = 30 μm or 20 μm, where partial overlap of WZs occurred, strain fluctuations intensified significantly, consistent with the results shown in Table 2. Such fluctuations suggest the presence of complex deformation in the DS direction of TC4 BM, which subjects the interfacial quartz BM to repeated impact-induced cracking, ultimately compromising the joint’s mechanical performance.

## 4. Conclusions

In summary, the direct bonding of quartz glass to the TC4 titanium alloy was performed using femtosecond laser welding, and the effects of pulse energy on microstructure, elemental distribution, and mechanical properties were investigated. The following conclusions were drawn:

1. The quartz glass-TC4 joint primarily consists of parallel laser-welded zones interspersed with BMs. Cracks appeared in specimens with +0.2 mm defocus distance or 20 μm weld spacing, which arises from micro-deformations generated during the melting and solidification of base metals. The diffusion of Si, O, and Ti elements occurs at the interface, indicating the sufficient flow and mixing of elements from the two base materials during the welding process. EDS results featured metallurgical bonding within WZs and van der Waals forces at WZ/BM interfaces.
2. The defocusing distance of the femtosecond laser predominantly influences the depth and phase composition of the WZs. As the focal position moves downward, increased energy absorption by the TC4 BM leads to significant expansion in both width and depth of the WZs, and subsequently influences effective bonding area and thermal mismatch conditions around the joint.
3. The shear strength of the joints initially increases and then decreases with increasing defocus distance and weld spacing. The maximum strength of 14.4 MPa was achieved at defocus distance of +0.1 mm and weld spacing of 40 μm. The XRD-RSM and GPA results demonstrate that variations in both parameters significantly influence the thermal expansion behavior of the BMs. Specifically, as the weld spacing decreases, the DS residual stress exhibit pronounced fluctuations. This phenomenon indicates that complex deformation occurs in the TC4 BM during post-weld cooling, which subjects the interfacial quartz BM to repeated impact-induced cracking, ultimately compromising the joint’s mechanical performance.

This study builds upon our previous work to further optimize the welding process between quartz glass and TC4 titanium alloy, providing technical recommendations for optical device fabrication in related fields. In addition, we analyze the relationship between joint residual strain and femtosecond laser weld spacing, providing valuable insights for optimizing femtosecond laser welding processes.

## Figures and Tables

**Table 1:** Chemical composition (wt. %) of the BMs.

Base Materials	Ti	Si	O	Al	V	Fe
TC4 alloy	87.4~91	<0.01	<0.16	5.5~6.8	3.5~4.5	<0.4
Quartz glass	/	46.5~46.7	53.2~53.4	/	/	0.01~0.03

**Table 2:** Parameters of the welding.

Groups of Samples	Power/W	Speed/mm·s^−1^	Frequency/kHz	Defocus Distance (f)/mm	Weld Spacing (d)/µm
1				−0.1	40
2				0	40
3				+0.1	40
4	3	50	10	+0.2	40
5				+0.1	20
6				+0.1	30
7				+0.1	50

**Table 3:** Results of the EDS point analysis.

Position	a	b	c	d
Element O (a.t.%)	45.22	36.50	25.50	20.50
Element Si (a.t.%)	34.31	26.81	18.81	3.81
Element Ti (a. t.%)	20.46	36.69	55.69	75.69
Secondary phases	SiO_2_, TiSi_2_	SiO_2_, TiSi_2_	TiSi_2_, TiO_2_	TiSi_2_, TiO_2_

**Table 4:** XRD-RSM test results.

Defocus Amount (f)/mm	Weld Spacing (d)/µm	Residual Stress of DLI/MPa	Residual Stress of DS/MPa
−0.1	40	32.1 ± 1.6	1.5 ± 1.3
0	40	65.4 ± 3.2	−2.9 ± 2.1
+0.1	40	73.2 ± 5.6	−4.8 ± 2.6
+0.2	40	96.3 ± 4.5	−5.5 ± 2.4
+0.1	20	74.5 ± 3.1	−8.3 ± 19.8
+0.1	30	76.6 ± 4.9	−6.1 ± 9.6
+0.1	50	69.5 ± 3.2	3.0 ± 1.2

## Data Availability

The original contributions presented in this study are included in the article. Further inquiries can be directed to the corresponding author.
